# Purple Urine Bag Syndrome: A Rare Phenomenon Managed in Primary Care

**DOI:** 10.7759/cureus.57620

**Published:** 2024-04-04

**Authors:** António P Pereira, Isabel Camarinha, Adriana Ferreira, Hugo Sevivas, Mariana Reis

**Affiliations:** 1 Family Medicine, USF Ruães, Unidade Local de Saúde de Braga, Braga, PRT

**Keywords:** obstipation, family doctor, primary care, case report, urinary catheter, urinary tract infection, purple urine bag syndrome

## Abstract

Purple urine bag syndrome (PUBS) is a rare manifestation of urinary tract infection (UTI) characterized by bluish-purple discoloration of urine, typically seen in patients with long-term urinary catheters. It occurs due to the bacterial metabolism of dietary tryptophan into indole, which is converted into indoxyl sulfate in the liver and then excreted in the urine, where it reacts with catheter materials under alkaline conditions. Risk factors include chronic constipation, advanced age, female gender, dehydration, and recurrent UTIs. *Morganella morganii* is one of the bacteria commonly implicated. Although usually benign, PUBS can signal underlying UTIs, especially in asymptomatic patients.

The case presented involves an 81-year-old woman with a history of urothelial carcinoma and a monoJ catheter since pelvic exenteration and ureterostomy. She presented with blue-colored urine in the collection bag; she was asymptomatic, but the urinalysis had leukocytes, nitrites and alkaline pH, and the uroculture was positive for* M. morganii*. Antibiotic therapy resolved the symptoms initially, but recurrence prompted further treatment and optimization of constipation management.

PUBS, although rare, poses risks, especially in vulnerable populations. Identification of risk factors and causative agents is crucial for effective treatment, typically involving catheter replacement, antimicrobial therapy, and constipation correction. Prevention focuses on minimizing catheter use, regular replacement, and hygiene optimization. Early recognition and management in primary care settings can prevent complications and reduce patient and caregiver distress.

In conclusion, PUBS is a visually evident condition that may serve as an early indication of UTI, particularly in patients with chronic catheterization. Treatment and prevention strategies should be tailored to individual risk factors to prevent the recurrence or persistence of the syndrome. Awareness among healthcare professionals and patients is essential for timely diagnosis and management. The presented case demonstrates the importance of primary care in managing complex conditions and highlights the close patient-physician relationship in such settings.

## Introduction

Purple urine bag syndrome (PUBS) is a rare manifestation of urinary tract infection (UTI) with alkaline pH, which predominantly occurs in patients with long-term urinary catheters and is characterized by a bluish-purple discoloration of the urine [1,2]. It arises due to the intestinal metabolism of dietary tryptophan into indole, a substrate that is transported to the liver, where it is converted into indoxyl sulfate. Urinary excretion of this compound promotes its transformation by bacteria with sulfatase/phosphatase activity. In an alkaline environment, the metabolism of indoxyl leads to the formation of indirubin (red pigment) and indigo (blue pigment), which precipitate and react with the synthetic materials of the catheter and urine bag, resulting in the bluish-purple coloration of the urine [3]. This phenomenon is more common in constipation situations because the increased intestinal transit time allows bacterial growth, promoting the conversion of tryptophan into indole. Other risk factors include advanced age, female gender, dehydration, use of catheter/urinary bags made of polyvinyl chloride (PVC), and recurrent UTIs [4]. The most commonly implicated bacteria are *Morganella morganii, Providencia stuartii, Providencia rettgeri, Klebsiella pneumoniae, Proteus mirabilis, Escherichia coli*, and *Pseudomonas aeruginosa* [5]. Although it is usually a benign syndrome, its prevalence has been increasing, and the underlying UTI can lead to serious consequences in patients with comorbidities [6]. Discoloration of urine in the urinary bag may be the only manifestation of a UTI in these patients, so it is important to pay proper attention to this alteration.

In this article, we present a case report that describes the clinical history and physical examination findings of an 81-year-old woman who was diagnosed with PUBS and was primarily managed in the Primary Care system.

This article aims to report the occurrence and the characteristics of this rare syndrome, with the intention of achieving early diagnosis and appropriate management, preventing possible complications, and reducing the stress caused to professionals, patients, and families in dealing with the unknown.

## Case presentation

The case presented concerns an 81-year-old female patient with a history of urothelial carcinoma (diagnosed in November 2022), in the context of which she underwent pelvic exenteration and ureterostomy, leaving her with a monoJ catheter. Since then, she has had nursing appointments at her primary care facility every three days for care of the ureterostomy.

**Figure 1:**
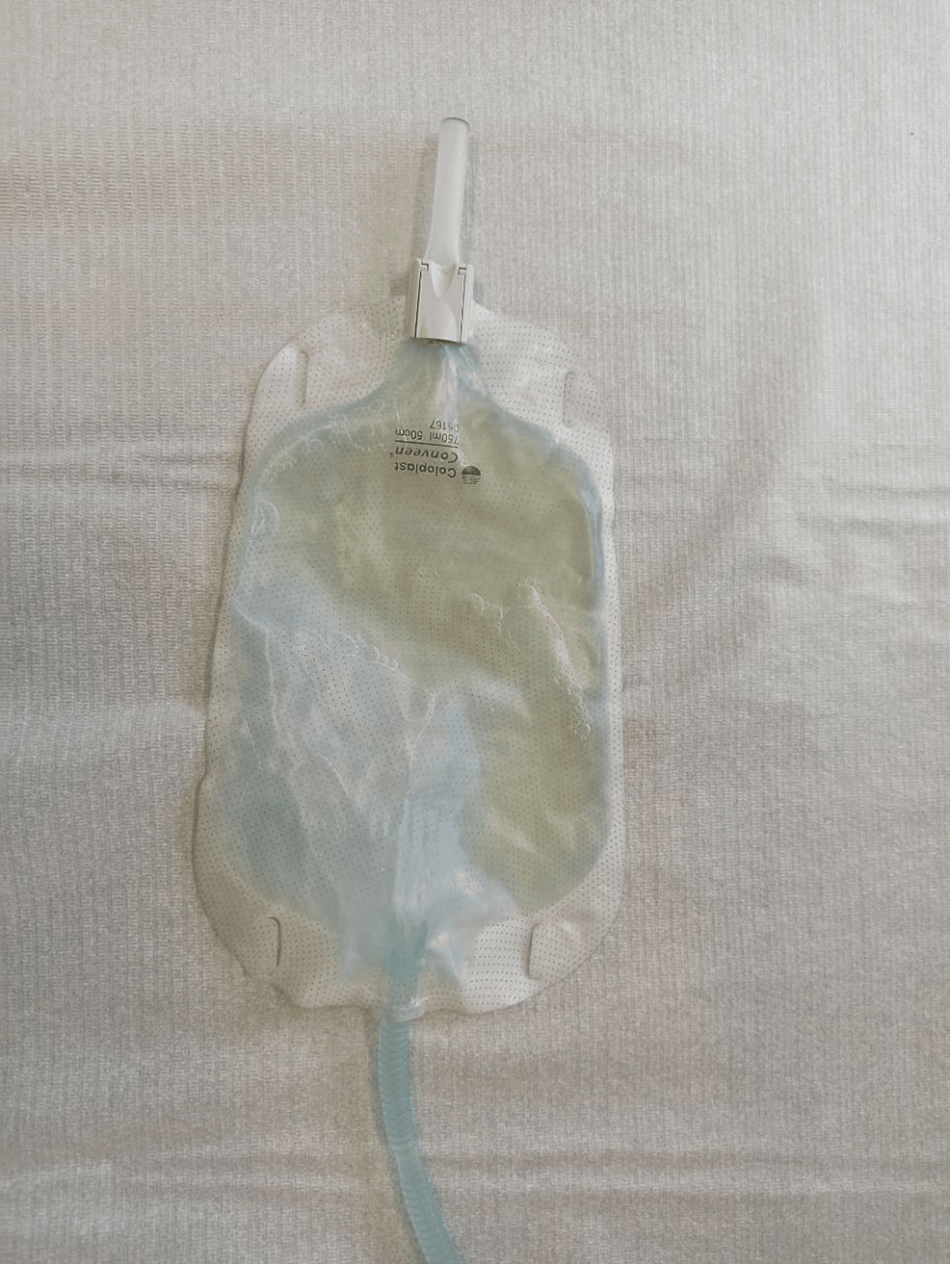
The patient’s collection bag, displaying blue-colored urine, when she first consulted her family doctor in November 2023

In November 2023, she consulted her family doctor because of blue-colored urine in the collection bag for the past seven days (Figure 1) - consent was obtained for all the pictures. At the time, the patient was asymptomatic, denying fever, vomiting, or associated back pain. In this context, urinary sediment and urine culture were requested by direct extraction from the ureterostomy catheter.

Two weeks later, the patient returned to her primary care facility, still with the urine color alteration, and brought the results of the requested tests. These showed the presence of leukocytes, nitrites, a pH of 7.5, as well as a positive urine culture, identifying the agent *M. morganii*. In this regard, and considering the antibiotic susceptibility test (AST), it was decided to initiate antibiotic therapy with trimethoprim + sulfamethoxazole for a period of seven days, considering the patient's asymptomatic condition and the impossibility of changing the monoJ catheter in that context. When asked about her intestinal transit, the patient mentioned being habitually constipated for several years. After the antibiotic therapy period, the urine in the collection bag returned to a clear yellow color.

**Figure 2:**
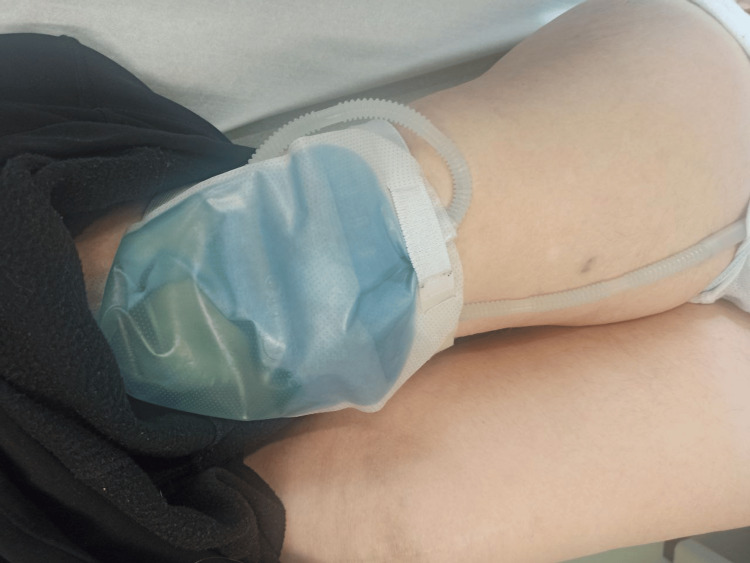
The patient’s collection bag, again with blue-colored urine, in January 2024

However, a month later, during one of the routine nursing appointments, the patient again presented with blue-colored urine in the collection bag (Figure 2). A new urine culture was collected, and she was again medicated with trimethoprim + sulfamethoxazole, this time for a period of 10 days. The urine culture was again positive, once more identifying *M. morganii*. In addition, the therapeutic approach to the patient's constipation was optimized and, since then, no new episodes have been reported.

## Discussion

PUBS is, indeed, a rare entity; however, it should not be ignored due to the detrimental effects it can have on the population in which it is most prevalent. Just like in the presented case, patients with chronic/permanent catheterization and bedridden elderly individuals, or those with multiple comorbidities, have an increased risk of developing UTIs [7]. In addition, several other factors contribute to the onset of PUBS, with chronic constipation being one of the most relevant, as was also observed in the reported case. Other risk factors present in this situation included advanced age, female gender, recurrent UTIs, and the presence of alkaline urine [1,4,5]. Another factor present in the described case was the identification of the bacterium *M. morganii* as the causative agent of the UTI, which is part of the group of bacteria associated with this syndrome, according to the literature [5].

Although the prognosis and evolution of this syndrome are usually favorable, it remains an alarming situation for the patient, family, and healthcare professionals, who are often unfamiliar with this phenomenon. Furthermore, there are cases described in the literature associated with significant morbidity and mortality [6], so it is essential to focus on its treatment and, especially, on its prevention. The medical treatment of PUBS depends on identifying the underlying cause - in most cases a UTI and/or constipation. Catheter replacement, correction of constipation, and antimicrobial therapy directed at the responsible agent are usually sufficient to resolve symptoms [8].

In the presented case, antibiotic treatment of the UTI with trimethoprim + sulfamethoxazole, recommended empirically by guidelines and according to the AST, and optimization of constipation were indeed sufficient for resolution. Prevention relies on the minimal possible use of catheters, their regular replacement (when removal is not possible), and optimized device hygiene. Prior correction of risk factors such as constipation also reduces the likelihood of this complication occurring [5,8].

## Conclusions

PUBS is a relatively unknown condition that results in striking changes in the color of urine in the collection bag or catheter in patients with chronic catheterization. The diagnosis is visually evident and may serve as an early indication of a UTI. Treatment involves catheter replacement and targeted antibiotic therapy, as well as control of risk factors, to prevent recurrence or persistence of this condition. In this regard, it is crucial to inform both healthcare professionals and family members about this phenomenon.

The particularity of the presented case was that all management, from detection, diagnosis, treatment, follow-up, and prevention of recurrences, was carried out in primary care, once again demonstrating the close relationship between patients and their family physicians.
